# Psychological symptoms in perimenarcheal adolescents: association with PCOS risk factors

**DOI:** 10.3389/fendo.2025.1551958

**Published:** 2025-06-12

**Authors:** Heidi Vanden Brink, Kathleen C. McCormick, Marla E. Lujan, Jane Chang, Lisa Ipp, Erika L. Mudrak, Anisah Alladeen, Hannah Lamar, Joy Y. Kim, Jane Mendle

**Affiliations:** ^1^ Department of Nutrition, Texas A&M University, College Station, TX, United States; ^2^ Institute for Advancing Health through Agriculture, Texas A&M AgriLife, College Station, TX, United States; ^3^ Department of Psychology, Cornell University, Ithaca, NY, United States; ^4^ Division of Nutritional Sciences, Cornell University, Ithaca, NY, United States; ^5^ Department of Pediatrics, Section of Adolescent Medicine, Weill Cornell Medicine, New York, NY, United States; ^6^ Cornell Statistical Consulting Unit, Cornell University, Ithaca, NY, United States

**Keywords:** PCOS, puberty, depression, anxiety, mental health, menarche

## Abstract

**Introduction:**

PCOS is linked with disproportionately high rates of depression and anxiety that significantly compromise quality of life and pose problems for treatment eligibility and adherence. The overarching objective of the proposed manuscript is to define the presence and severity of psychological symptoms in peri-menarcheal adolescents, and their association with well-described risk-factors for future PCOS.

**Methods:**

Fifty-two pre- and early post-menarcheal participants underwent a non-fasting blood draw to measure reproductive hormones (Anti-Mullerian Hormone (AMH), Luteinizing Hormone (LH), Follicle Stimulating Hormone (FSH), Total Testosterone, Sex Hormone Binding Globulin (SHBG) and HbA1c, anthropometry, menstrual history (if post-menarcheal), and a series of surveys to evaluate depression (CES-DC), anxiety (MASC) and, as a novel approach, rumination, which is a transdiagnostic psychological process and early prodromal risk for psychological disorders. Parents/legal guardians completed a demographics survey. Random Forest analysis was used to predict depression, anxiety, and rumination from a predetermined set of variables in this participant sample.

**Results:**

The overall R^2^ for depression, anxiety, and rumination from the random forest model were 0.557, 0.555, and 0.597, respectively, suggesting overall good explanatory power for psychological outcomes. Parent education (Portion Sum of Squares (SS) = 11.4%) followed by AMH (Portion SS = 10.9%) and waist-hip-ratio (WHR) (Portion SS = 9.2%) were the most important variables in predicting depression. LH: FSH ratio was the most important variable in the dataset used to differentiate participants along the observed anxiety score continuum (Portion SS = 0.112 (11%) followed by HbA1c (Portion SS = 8.1%) and WHR (Portion SS = 7.9%). SHBG was the most frequently identified variable to differentiate participants reporting rumination (Portion SS = 13.3%) followed by Free Androgen Index (Portion SS = 6.9%) and WHR (Portion SS = 6.9%). Adolescents at high risk for progression to PCOS may already experience psychological vulnerabilities prior to a clinical diagnosis or full manifestation of PCOS. Our study findings highlight PCOS as a lifelong, multifaceted health condition with ramifications earlier than commonly documented.

## 1 Introduction

Polycystic ovary syndrome (PCOS) is a highly prevalent endocrine condition affecting approximately 1 in 10 females (1–3), and diagnosed by the presence of hyperandrogenism, menstrual irregularity, and in adults enlarged, polycystic ovaries or elevated Anti-Mϋllerian Hormone (AMH) (4). PCOS is a multi-system disease whose pathophysiology is intertwined with significant cardiometabolic abnormalities – including obesity, Type 2 diabetes, hypertension, dyslipidemia, coronary heart disease, and stroke – resulting in disproportionately higher rates of disability and health-related unemployment (4–8). Because PCOS emerges during adolescence, there is a growing scientific and medical consensus that early intervention is crucial for mitigating the lifelong chronic disease burden.

Inexplicably high rates of depression and anxiety have been documented in adolescents and adults with PCOS (9–12), even after consideration of comorbid conditions also closely tied to psychopathology (13). These high rates of psychopathology are poorly understood despite being a crucial consideration for both successful interventions and overall quality of life; mental health comorbidities are known to compromise treatment adherence for both pharmacological and behavioral interventions for PCOS (14, 15) and further increase the risk of cardiometabolic disease among women with PCOS (16). Yet paradoxically, several PCOS interventions (including surgical, pharmacologic, and lifestyle interventions) list psychological comorbidities as exclusionary (17–19), which limits the inference regarding the effectiveness of new and existing interventions and therefore restricts evidence-based options to a subset of those living with PCOS.

Whether psychological symptoms emerge before, after, or concurrent with the diagnostic features of PCOS is neither clear nor well studied. Depression and anxiety are often identified and researched following the diagnosis of PCOS, a disease which has no cure and often is not recognized until after at least a year of actively seeking a diagnosis (20). While the diagnostic experience itself may play an important role in exacerbating pre-existing depression and anxiety, this approach confounds the question of time course and the physiological relationship between PCOS and psychopathology. Thus, whether investigators are measuring psychological vulnerabilities inherent to PCOS – versus the burden of a disorder known to impact quality of life, employment, finances, and childbearing – is impossible to disentangle in the existing literature.

To begin to address this knowledge gap, we conducted a pilot study of pre- and post-menarcheal adolescents to determine whether endocrine or clinical features of PCOS, physical changes of puberty, or age best predicted the severity of depression, anxiety, and rumination. We hypothesized that symptoms of PCOS would emerge as early predictors of PCOS; however we used an unbiased analytical approach without setting priority predictors *a priori.* While depression and anxiety are well-studied in regard to PCOS, rumination has been considered less frequently. Rumination refers to the tendency to cope with negative emotions by thinking repetitively and passively about distress and the causes and consequences of this distress (21). Ruminative coping has been documented in children as young as 8 years of age (22), rises sharply at puberty (23), and is both a correlate and robust prodromal indicator of depression and anxiety (24). Because rumination often precedes the onset of depression and anxiety, its inclusion in this study offers a chance to understand early connections between psychological states and features of PCOS.

## 2 Methods

### 2.1 Participants

Fifty-two children and adolescents (9–15 years) were enrolled in a cross-sectional study across two sites (Upstate New York; Cornell University, New York City; Weill Cornell). Recruitment was open to all-comers who met eligibility criteria. Recruitment in New York City occurred mainly through an adolescent medicine clinic, although community and employee-based recruitment tools were also employed. Across both sites, study information was distributed using social media, listservs, and flyers posted in clinical and community settings. Participants were recruited based on one of three categories: i) premenarcheal and at least Tanner Stage 3 for either pubic hair or breast development based on self or parent report (Cornell) or physician report (Weill Cornell), ii) within 2 years of menarche with regular menstrual cycles or iii) within 2 years of menarche with irregular menstrual cycles. Regular menstrual cycles were defined as 3 successive cycles 21–45 days apart and irregular cycles were defined as having at least 1 of 3 successive cycles <21 days or >45 days apart (4). All adolescents within the first year of menarche were classified as irregular (4). Exclusion criteria included significant delays in development that influenced social, cognitive, emotional, or behavioral functioning, pregnant or breastfeeding, history of chronic disease (i.e. type 1 or 2 diabetes), precocious puberty, or currently taking medications that affect reproductive functioning (e.g. hormones, metformin, or some anti-seizure medications).

### 2.2 Ethics

The study was approved by ethics review boards at Cornell University and Weill Cornell Medicine. All procedures in this study were carried out in accordance with the World Medical Association’s Declaration of Helsinki. Participant assent and parental/legal guardian permission were obtained after confirming that the participant was eligible for participation and before study procedures were initiated.

### 2.3 Clinical study procedures

Participants attended one non-fasting study visit and underwent the following researcher-guided standardized interviews: participant menstrual history (if post-menarcheal), participant medical history, family medical history, standardized assessment of acne presence and severity (25), and hirsutism scoring with visual aids using the modified Ferriman-Gallwey scoring system (26). Anthropometric measures of weight, height, waist and hip circumference, blood pressure, and heart rate were obtained using standard procedures. A non-fasting blood draw was collected into serum separator and EDTA tubes via antecubital venipuncture. Whole blood, serum, and plasma was processed per protocol and frozen immediately at -80C for long-term storage.

### 2.4 Psychological measures

#### 2.4.1 Depressive symptoms

The Center for Epidemiologic Studies Depression Scale for Children (CES-DC (27);) is a 20-item, self-report measure of depressive symptoms experienced in the past week. Each item is rated on a scale where 0 =*not at all* and 3 = *a lot*, with higher scores indicating a greater frequency and severity of depressive symptoms. A score of 15 is typically used to signify a clinically relevant level of symptoms (27). Scores in this sample ranged from 2 to 46 (*M=*22.7, *SD=*12.8). Cronbach’s α = 0.80.

#### 2.4.2 Anxiety symptoms

The Multidimensional Anxiety Scale for Children – 2^nd^ Edition (28) is a 50-item self-report questionnaire assessing physical, cognitive, behavioral and affective symptoms of different forms of anxiety, including worry, panic, social anxiety, separation anxiety, and harm avoidance. Each item is rated on a scale where 0 = *never* and 3 = *often*, with higher scores indicated a greater frequency and severity of anxiety symptoms. Scores in this sample ranged from 17-99 (*M=*56.8, *SD=*21.7). Cronbach’s α = 0.94.

#### 2.4.3 Rumination

Rumination was assessed using the Ruminative Response Scale of the Children’s Response Styles Questionnaire (22, 29), a 13-item measure of self-focused, cognitive responses to feelings of sadness modeled after the adult version of the Response Styles Questionnaire (30). Each item is scored on a scale where 0 = *almost none of the time* and 3 = *almost all of the time*, with higher scores indicating a greater tendency towards rumination. In this sample, scores ranged from 2-39 (*M =* 18.3, *SD =* 9.5). Internal consistency was good (Cronbach’s α = 0.91).

### 2.5 Biochemical assays

Serum androgens were evaluated by Brigham and Women’s Research Assay Core Laboratory using liquid chromatography with tandem mass spectrometry (Boston, MA; interassay CV 6.4%). AMH was measured using the picoAMH enzyme-linked immunosorbent assay (Motive Biosciences Inc., Webster, TX). The remaining analytes were shipped to the Human Nutritional Chemistry Service Laboratory at Cornell University for immunoassay analysis (Immulite 2000, Siemens Medical Solutions Diagnostic, Deerfield, IL). Free Androgen Index (FAI) was calculated as Total Testosterone/Sex Hormone Binding Globulin (SHBG) x 100% (31). FAI is a surrogate for free testosterone and an accepted marker for biochemical hyperandrogenism per the 2023 International Guidelines for PCOS (4).

### 2.6 Statistical analysis

REDCap^®^ (Research Electronic Data Capture) was used to securely store study data. We conducted Random Forest analysis, which is an ensemble machine learning technique that aggregates a large number of decision trees (32) to optimally predict an outcome (e.g., depression score). A particular strength of this methodology is its ability to handle a large number of covariates per observation and considers complex interactions (32), which we deemed appropriate for our dataset, which included predictors spanning puberty, body habitus, social demographics, and PCOS. A decision tree iteratively searches for predictors on which to divide the dataset so that the residual sum or squares is minimized in each group. It then repeats the process recursively, dividing each subgroup by different predictors until an optimal tree is determined. While analysis using a single decision tree results in models that are biased and prone to overfitting, a random forest analysis generates many different trees that are each trained using a bootstrapped random sample from the original data set (to avoid overfitting) using subsamples of the set of predictor variables (to decorrelate the set of trees). The random forest procedure then averages the predictions of each tree, resulting in a better-fitting model with a lower variance and prediction error than that of any single tree. Another useful angle of a random forest analysis is the ability to calculate the relative variable importance to provide an assessment of each variable’s contribution to the predictive power of the model.

We used JMP Pro (Version 17) to conduct three separate random forest models, one each for the aggregate score for depressive symptoms, anxiety symptoms, and rumination. For each psychological outcome, we trained the model using 1000 trees, requesting 10 minimum splits per tree with a minimum subgroup size of 5. The minimum number of terms (variables) sampled per split was set to 7 for depression, 10 for anxiety, 10 for rumination. These values were arrived at after assessing several options using the tuning design table feature of JMP. The results of the 1000 trees per outcome are aggregated and summarized by listing the rank order of all variables included, from most important to least important, including the number of times each variable was used in a tree (split number). **Supplementary Table 1** presents the full list of variables selected for inclusion in the model; these variables were selected based on diagnostic features of PCOS, known risk factors or PCOS, or their identification in previous research as predictive of psychological disorders in adolescence (e.g., Tanner Staging, sociodemographic variables).

## 3 Results

### 3.1 Participant characteristics

Participant demographics by reproductive age are depicted in **Table 1**. Participants were on average between 11–12 years old, most did not have a family history of PCOS or known infertility and most (77%) had never taken psychotropic medications. On average, participants were within a normal BMI and glycemic control (**Table 1**); however, 23% of participants met criteria for obesity and one participant had an HbA1c of 5.7%, which meets criteria for pre-diabetes (33). Overall, we recruited a diverse sample of youth (65% White, 21% Black/African-American, and 8% Asian, 27% Hispanic; percents do not equal 100 as participants were allowed to select all demographics that applied to them). Differences in depression anxiety, and rumination scores did not differ across pre-menarcheal and first- and second- post-menarcheal age groups (p>0.05).

**Table 1:** Participant characteristics.

	Pre-Menarcheal		First Year Post-Menarche		Second Year Post-Menarche	
	n=16		n=24		n=12	
	Mean	SD	Mean	SD	Mean	SD
Participant Characteristics						
Age (yrs)	11.7	1.2	12.5	1.3	12.7	1.4
Age at menarche (yrs)	–	–	12.1	1.2	11.2	1.2
Gynecological age (yrs)	–	–	0.4	0.3	1.5	0.3
Family History of PCOS or Infertility						
Yes (n)	2		5		0	
No (n)	14		19		12	
Use of psychotropic medication medications						
Ever (n)	0		5		1	
Never (n)	16		19		11	
Psychological Survey Scores						
CESD Score (Sum)	21	10.4	24	14.7	22	12.3
MASC Score (Sum)	53	26.0	60	18.8	56	22.1
Rumination Score (Sum)	16	8.8	21	10.9	17	6.5
Metabolic Health						
BMI Percentile (for age, height)	66.3	32.4	63.5	27.0	72.0	25.7
<85th %ile (n)	9		16		8	
85th - 94.9th %ile (n)	3		2		2	
>95th %ile (n)	4		6		2	
Waist-Hip-Ratio	0.8	0.1	0.8	0.1	0.8	0.1
HbA1c (%)	5.1	0.3	5.2	0.2	5.1	0.2
SHBG (nmol/L)	62.2	23.8	48.7	22.7	61.3	30.6
Clinical Features of Reproductive Maturity						
Sexual Maturity Scale - Breast Development	2.9	0.5	3.5	0.7	4.0	0.7
Sexual Maturity Scale - Pubic Hair	3.0	1.0	3.8	0.8	3.9	1.0
Dysmenorrhea						
Reproductive Health						
LH (mIU/mL)	1.9	1.8	4.3	3.1	7.7	11.8
FSH (mIU/mL)	3.7	1.6	4.9	2.1	4.1	2.5
LH: FSH Ratio	0.5	0.4	0.9	0.6	1.7	1.2
Estradiol (pg/mL)	29.3	19.6	45.3	36.1	62.1	59.0
Diagnostic Features of PCOS						
Hirsutism Score	0.7	0.9	2.1	3.2	4.5	5.9
Acne Grading Score	1.0	0.6	1.5	0.7	1.6	1.0
AMH (ng/mL)	4.0	2.7	3.4	3.0	6.4	3.6
Total Testosterone (ng/dL)	14.8	9.0	19.7	8.4	22.0	6.7
Free Androgen Index (%)	0.9	0.5	1.7	0.9	1.4	0.5

### 3.2 Variable importance for depression scores

**Figure 1:**
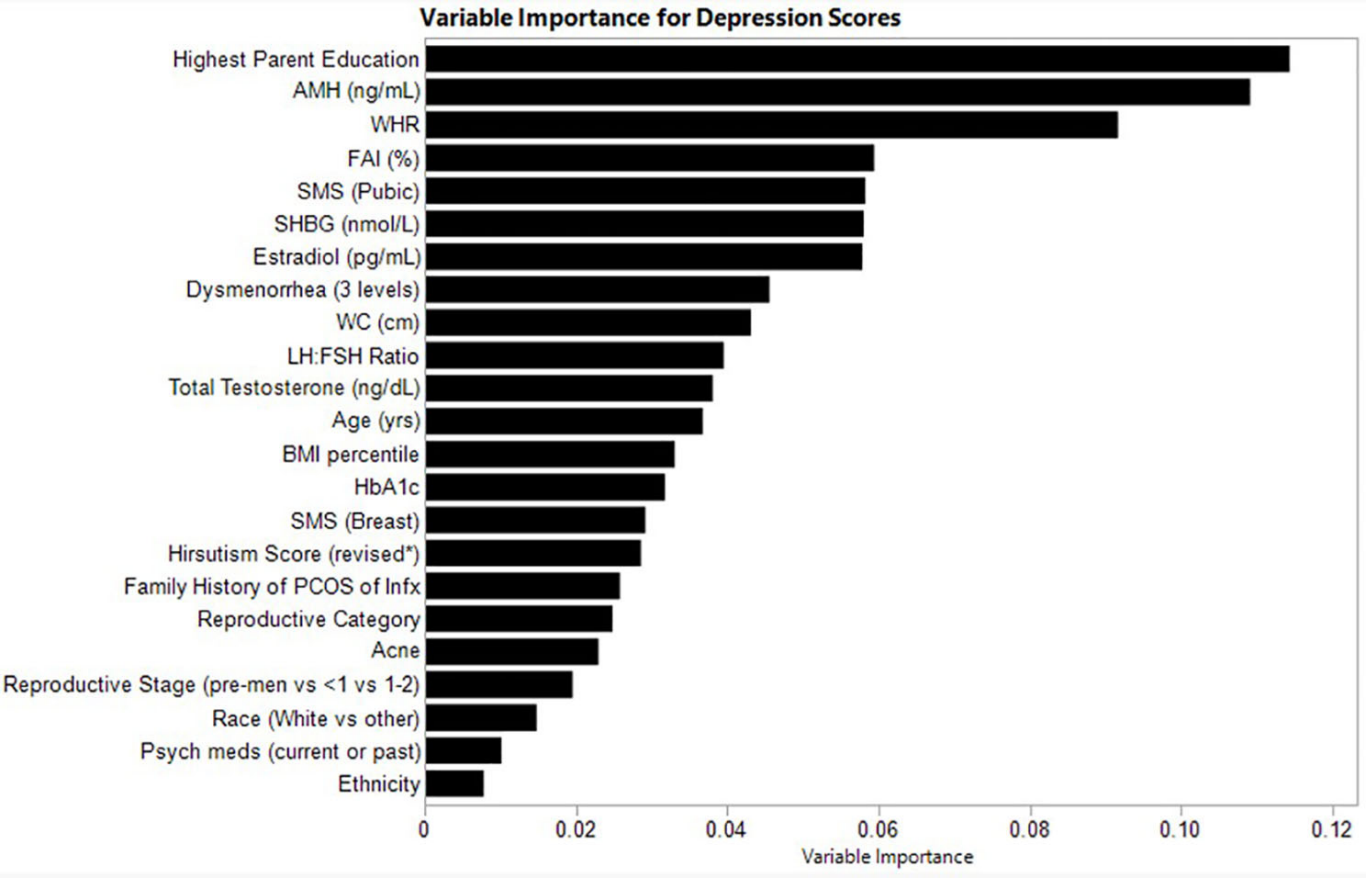
Relative variable importance scores for depression in pre- and post-menarcheal adolescents. Variables are ranked in order of importance, with overall importance (sum of squares) on the x-axis. AMH, Anti-Mullerian Hormone; BMI, Body Mass Index; FAI, Free Androgen Index (%); SHBG, Sex Hormone Binding Globulin; SMS, Sexual Maturity Scale; WC, waist circumference; WHR, Waist-Hip-Ratio. For a detailed explanation of variables please see **Supplementary Table 1**.

The relative variable importance scores for the observed depression score model are presented in **Figure 1**. Number of splits per variable and sum of squares presented in **Table 2**. Of the 52 participants, 51 were included in the analysis (n=1 did not complete the depression score survey). The overall R^2^ for this random forest model was 0.557 and overall Root Mean Square Error (RMSE) = 10.21, suggesting overall good explanatory power for depression scores in this sample using the variables. Parent education (Portion Sum of Squares = 0.114; **Table 2**), AMH (Portion Sum of Squares = 0.109; **Table 2**), and waist-hip-ratio (Portion Sum of Squares = 0.092; **Table 2**) emerged as top variables (**Figure 1**). Collectively, these three variables represented 0.32 (32%) of the total Sum of Squares in depression scores.

**Table 2:** Variable contributions for depression.

Variable	Number of Splits	Sum of Squares (SS)	Portion of SS
Highest Parent Education	414	314.86	0.114
AMH (ng/dl)	494	300.51	0.109
WHR	473	252.36	0.092
FAI (%)	352	163.65	0.059
SMS (Pubic)	308	160.37	0.058
SHBG (nmol/L)	372	159.89	0.058
Estradiol (pg/mL)	364	159.31	0.058
Dysmenorrhea (3 levels)	331	125.52	0.046
WC	313	118.76	0.043
LH: FSH Ratio	319	108.88	0.040
TT (ng/dL)	311	104.87	0.038
Age (yrs)	292	101.21	0.037
BMI (%ile for age)	292	91.08	0.033
HbA1c (%)	271	87.51	0.032
SMS (Breast)	222	80.33	0.029
Hirsutism Score	239	78.69	0.029
Family History of PCOS/Infertility	130	71.09	0.026
Reproductive Category	244	68.38	0.025
Acne Score	188	63.27	0.023
Reproductive Stage (3 levels)	226	53.85	0.020
Race (White vs Other)	170	40.77	0.015
Psych Med Use (current or past)	65	27.99	0.010
Ethnicity	122	21.50	0.008

The number of splits refers to the number of times each variable was used in the random forest trees to explain depression scores within the sample. Sum of Squares (SS) reports cumulatively, the degree to which each variable helped to improve the predictive power of the random forest model. AMH, Anti-Mullerian Hormone; BMI, Body Mass Index; FAI, Free Androgen Index (%); SHBG, Sex Hormone Binding Globulin; SMS, Sexual Maturity Scale; WC, waist circumference; WHR, Waist-Hip-Ratio. For a detailed explanation of variables please see **Supplementary Table 1**.

Heatmaps to highlight the non-linearity of interactions between the top three covariates for depression scores are depicted in **Supplementary Figure 1**. The heatmaps show the predicted values of depression scores by the top predictor variables, also considering all other covariates included in the random forest model. White squares reflect no observations along the x- and y-axis variables and therefore no data. The relationship between predicted and observed psychological outcomes are depicted in **Supplementary Figure 4**.

### 3.3 Variable importance for anxiety scores

**Figure 2:**
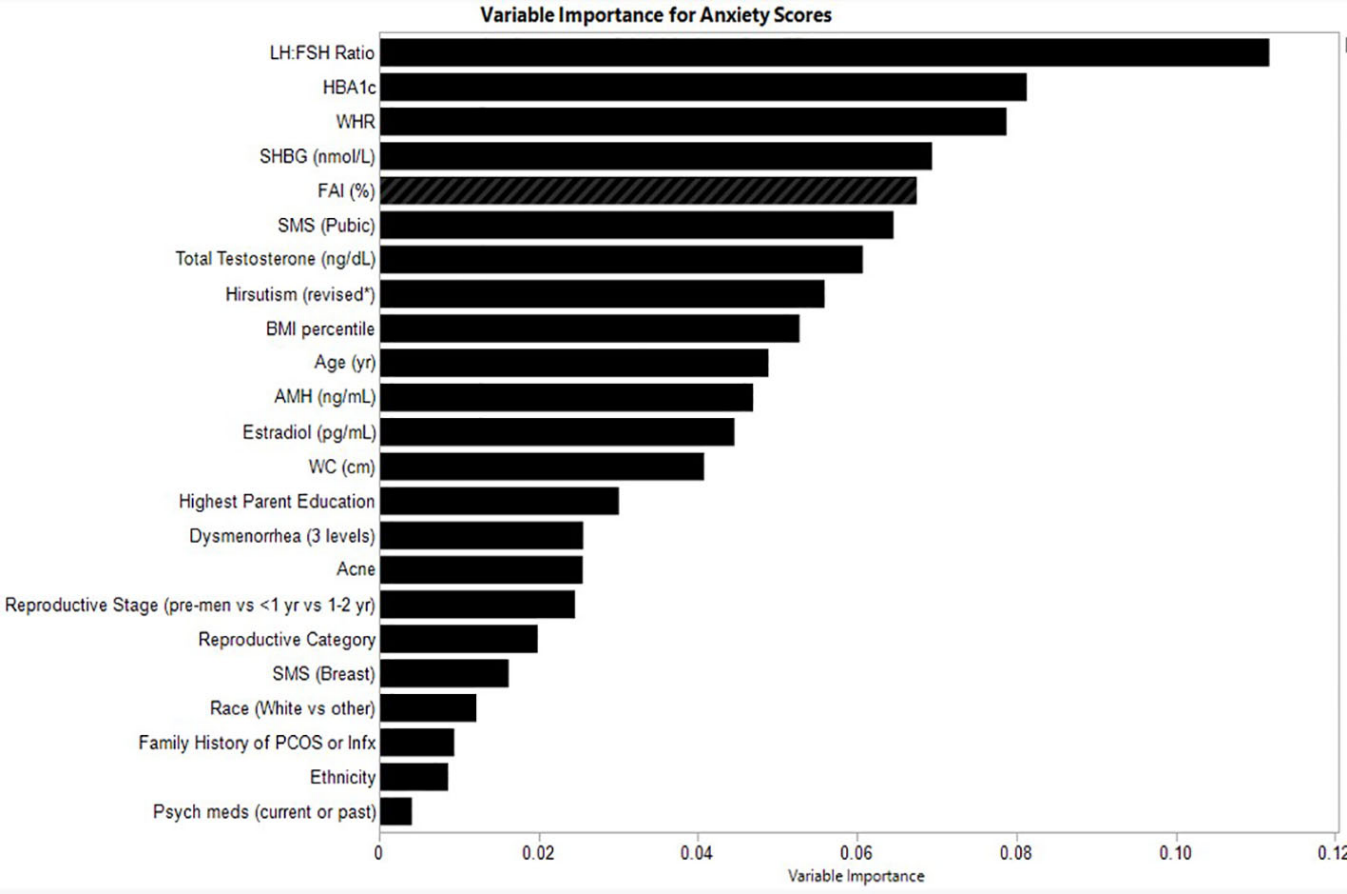
Relative variable importance scores for anxiety in pre- and post-menarcheal adolescents. Variables are ranked in order of importance, with overall importance (sum of squares) on the x-axis. AMH, Anti-Mullerian Hormone; BMI, Body Mass Index; FAI, Free Androgen Index (%); SHBG, Sex Hormone Binding Globulin; SMS, Sexual Maturity Scale; WC, waist circumference; WHR, Waist-Hip-Ratio. For a detailed explanation of variables please see **Supplementary Table 1**.

The relative variable importance scores for the observed anxiety score model are presented in **Figure 2**. Number of splits per variable and sum of squares presented in **Table 3**. Of the 52 participants, 49 were included in the analysis (n=3 did not complete the anxiety score survey). The overall model fit using random forest trees based on the included variables was R^2^ = 0.555 and overall RMSE = 14.4, again suggesting good explanatory power of anxiety scores in this sample. The LH: FSH ratio was the most important variable in the dataset used to differentiate participants along the observed anxiety score continuum, explaining 0.112 (11%) of the total Sum of Squares for anxiety scores. HbA1c and waist-hip ratio exhibited similar explanatory power (Portion Sum of Squares = 0.081 and 0.079, respectively; **Table 3**). Heatmaps highlighting non-linearity of interactions between the top three covariates for anxiety scores are depicted in **Supplementary Figure 2**; the relationship between predicted and observed psychological outcomes presented in **Supplementary Figure 4**.

**Table 3:** Variable contributions for anxiety.

Variable	Number of Splits	Sum of Squares (SS)	Portion of SS
LH: FSH Ratio	521	944.62	0.112
HbA1c (%)	445	687.40	0.081
WHR	413	665.77	0.079
SHBG (nmol/L)	410	586.77	0.069
FAI (%)	367	570.62	0.067
SMS (Pubic)	352	545.93	0.065
TT (ng/dL)	390	513.22	0.061
Hirsutism Score	303	472.56	0.056
BMI (%ile for age)	345	445.97	0.053
Age (yrs)	384	413.15	0.049
AMH (ng/dl)	373	396.89	0.047
Estradiol (pg/mL)	314	377.00	0.045
WC	313	344.69	0.041
Highest Parent Education	264	254.23	0.030
Dysmenorrhea (3 levels)	242	216.58	0.026
Acne Score	207	216.14	0.026
Repro By Gyn Yr	258	208.03	0.025
Reproductive Category	215	168.00	0.020
SMS (Breast)	166	137.32	0.016
Race (White vs Other)	146	103.22	0.012
Family History of PCOS/Infertility	82	79.51	0.009
Ethnicity	118	73.17	0.009
Psych Med Use (current or past)	55	34.73	0.004

The number of splits refers to the number of times each variable was used in the random forest trees to explain anxiety scores within the sample. Sum of Squares (SS) reports cumulatively, how much each variable helped in reducing the unexplained variability in depression scores. AMH, Anti-Mullerian Hormone; BMI, Body Mass Index; FAI, Free Androgen Index (%); SHBG, Sex Hormone Binding Globulin; SMS, Sexual Maturity Scale; WC, waist circumference; WHR, Waist-Hip-Ratio. For a detailed explanation of variables please see **Supplementary Table 1**.

### 3.4 Variable importance for rumination scores

**Figure 3:**
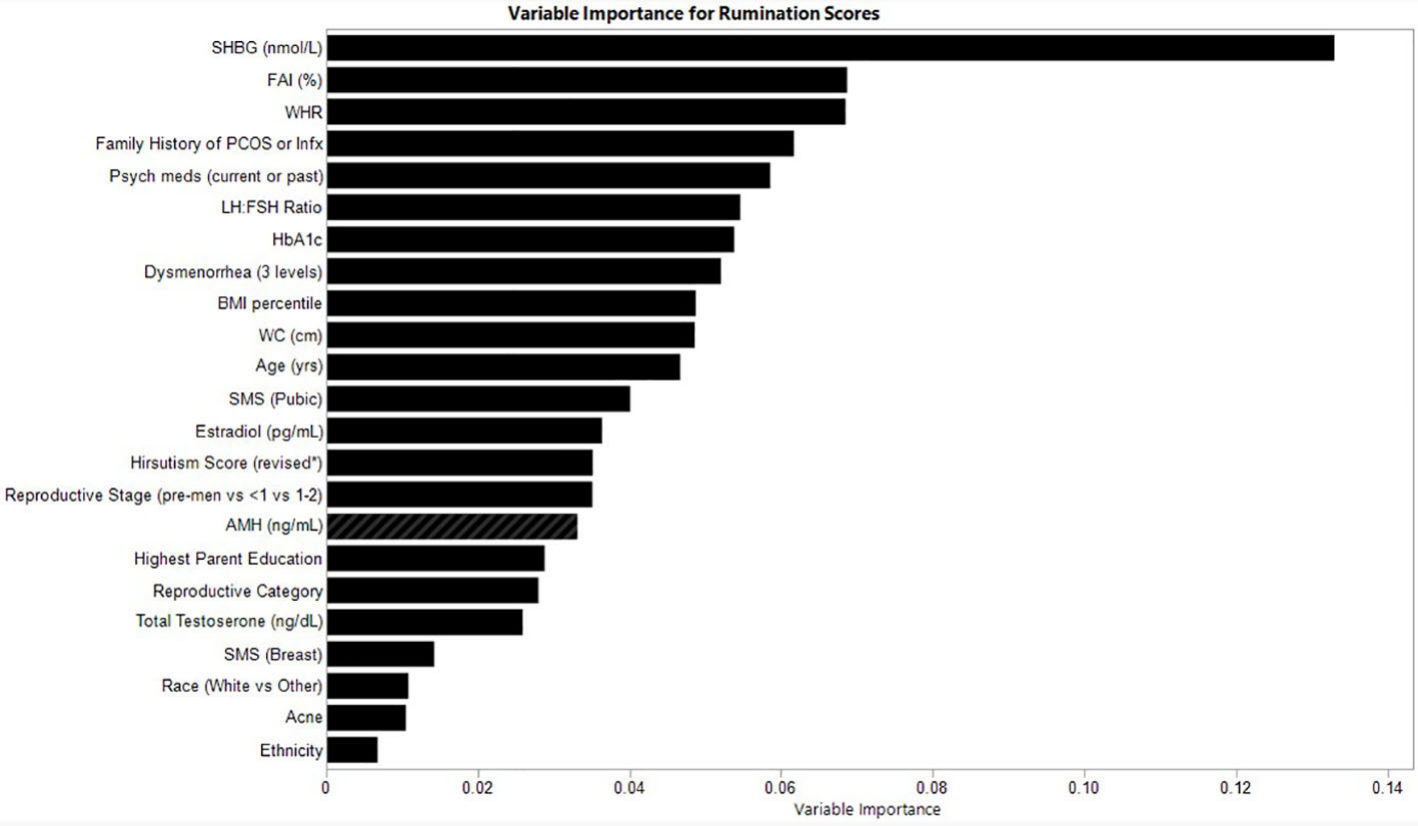
Relative variable importance scores for rumination in pre- and post-menarcheal adolescents. Variables are ranked in order of importance, with overall importance (sum of squares) on the x-axis. AMH, Anti-Mullerian Hormone; BMI, Body Mass Index; FAI, Free Androgen Index (%); SHBG, Sex Hormone Binding Globulin; SMS, Sexual Maturity Scale; WC, waist circumference; WHR, Waist-Hip-Ratio. For a detailed explanation of variables please see **Supplementary Table 1**.

The relative variable importance scores for the observed rumination score model are presented in **Figure 3**. Number of splits per variable and sum of squares are presented in **Table 4**. The overall model fit for rumination scores based on the included variables was good at R^2^ = 0.597, RMSE = 5.99. In predicting rumination, SHBG was the most frequently identified variable in the random forest trees to differentiate participants reporting rumination (Portion Sum of Squares = 0.13; **Table 4**). The next most important variable was FAI, which uses SHBG in its calculation (Portion Sum of Squares = 0.069), and waist-hip-ratio (Portion Sum of Squares = 0.069). Heatmaps highlighting non-linearity of interactions between the top three covariates for rumination scores are depicted in **Supplementary Figure 3**.

**Table 4:** Variable contributions of rumination scores.

Variable	Number of Splits	Sum of Squares (SS)	Portion of SS
SHBG (nmol/L)	597	236.661	0.133
FAI (%)	416	122.284	0.069
WHR	446	121.933	0.069
Family History of PCOS/Infertility	178	109.797	0.062
Psych Med Use (current or past)	182	104.214	0.059
LH: FSH Ratio	399	97.195	0.055
HbA1c (%)	406	95.755	0.054
Dysmenorrhea (3 levels)	379	92.625	0.052
BMI (%ile for age)	361	86.752	0.049
WC	358	86.523	0.049
Age (yrs)	390	83.101	0.047
SMS (Pubic)	290	71.356	0.040
Estradiol (pg/mL)	303	64.760	0.036
Hirsutism Score	317	62.545	0.035
Repro By Gyn Yr	313	62.448	0.035
AMH (ng/dl)	336	58.995	0.033
Highest Parent Education	289	51.278	0.029
Reproductive Category	275	49.793	0.028
TT (ng/dL)	280	46.133	0.026
SMS (Breast)	189	25.340	0.014
Race (White vs Other)	149	19.265	0.011
Acne Score	170	18.712	0.011
Ethnicity	121	12.072	0.007

The number of splits refers to the number of times each variable was used in the random forest trees to explain rumination scores within the sample. Sum of Squares (SS) reports cumulatively, how much each variable helped in reducing the unexplained variability in depression scores. AMH, Anti-Mullerian Hormone; BMI, Body Mass Index; FAI, Free Androgen Index (%); SHBG, Sex Hormone Binding Globulin; SMS, Sexual Maturity Scale; WC, waist circumference; WHR, Waist-Hip-Ratio. For a detailed explanation of variables please see **Supplementary Table 1**.

## 4 Discussion

The International Guideline for PCOS advises routine screening for depression and anxiety, recognizing the established burden of psychological well-being in women and adolescents with PCOS (4). In the present study, we sought to determine whether known predictors or early indicators of future PCOS among pre- and early post-menarcheal adolescents were associated with the degree of depression, anxiety, and rumination symptoms. Early endocrine predictors of PCOS and metabolic dysfunction – notably insulin resistance, hyperandrogenism, and central adiposity – consistently emerged as top variables in predicted depression, anxiety, and rumination scores. These results suggest that psychopathology may emerge early in PCOS, even before a clinical diagnosis.

The mechanistic basis for psychopathology in PCOS is poorly understood. Numerous connections have been proposed, including abnormalities in the hypothalamic-pituitary-adrenal axis, the emotional toll of infertility, chronic inflammation, and chronic cardiovascular problems secondary to PCOS (34). Yet none of these explanations have been consistently supported within the literature – and most would be unlikely to explain the established elevations of psychopathology in adolescents with PCOS. Our results align with several lines of human and pre-clinical evidence supporting a shared pathophysiology between PCOS and psychopathology. For example, the relevance of insulin receptor signaling in the brain was demonstrated in a brain-specific insulin receptor knockout mouse model establishing the importance of insulin signaling for dopamine turnover, reduction of which was associated with depression and anxiety (35). Likewise, rodent and sheep models to study genetic and epigenetic transmission of PCOS demonstrate that offspring of maternal PCOS animals exhibit frequent and persistent anxiety in offspring (36, 37).

The majority of literature on depression and anxiety in PCOS has been conducted following a prolonged diagnostic experience (20) and accompanied by increased awareness of PCOS-related health concerns (such as infertility, unwanted hair growth, weight management, risk of Type 2 Diabetes) and other long-term comorbidities (38, 39). The present study represents a novel, exploratory analysis in children and adolescents, most of whom were too young to be evaluated for PCOS (4) but may already have emerging symptoms of PCOS or evidence of reproductive dysfunction consistent with a trajectory towards PCOS. The alignment of PCOS correlates with psychological outcomes in our data suggest that the psychological challenges associated with PCOS begin even earlier than previously thought and may be part of the pathogenesis of the condition.

Notably, PCOS-related variables were more closely aligned with psychological outcomes than pubertal development, including menarche and Tanner Staging. This is surprising, given the robust research literature documenting rises in psychopathology in youth at puberty. This suggests there may be an early divergence in mental health symptoms for youth at risk for PCOS, over and above the well-established and expected rise in psychological distress during this window of development (40). Ultimately, this underscores the importance of continued research into the interdependence between reproductive maturation, PCOS pathogenesis, and psychological well-being.

In addition to endocrine correlates of PCOS and insulin resistance, parent education emerged as a top variable for depression, with higher parental education associated with more severe symptoms of depression. In general, the reverse pattern has been documented in the research literature to date (41, 42). Although interesting, we suspect this may be an artifact of our recruitment timeline: we launched this study during the height of the COVID-19 global pandemic. The COVID-19 pandemic associated shut-downs and disruptions were marked by increased depression among youth generally and females from socioeconomic backgrounds similar to our Upstate NY participants specifically (43). Our assumption is that it is unlikely associations of higher parental education and depressive symptoms would persist were data collection to be repeated during a less volatile time period.

Unique to the present study was our inclusion of rumination. Rumination is a familiar risk within psychological science, associated with both the onset and exacerbation of internalizing disorders. It is considered *transdiagnostic*, because it is linked with multiple psychological disorders versus just one, and it is malleable in evidence-based paradigms in both youth and adults (44, 45). Our findings confirm that, like depression and anxiety, rumination is also strongly connected to endocrine indicators and known risks for PCOS. Because rumination often precedes the onset of these disorders, clinicians and health practitioners may find assessments of rumination beneficial when treating or evaluating PCOS risk.

Our study holds several strengths. First, our choice to target early adolescence is unusual, as participants have not been diagnosed with PCOS and some have not even reached menarche. These results confirm that the interdependence of psychopathology with endocrine indicators of PCOS is present far earlier than generally discussed and reaffirms the value of a lifespan approach to PCOS. Second, in an effort to take an unbiased approach to identifying variables of interest for psychological outcomes we did not use linear models or test mean differences, which are more common and relevant for case-control designs (10, 12, 34). Instead, we employed a sophisticated, machine learning approach. As a result, analyses reveal a collection of traits – potentially representing an early pre-PCOS phenotype – that may be connected to greater symptoms of depression, anxiety, and rumination. While these analyses do not provide insight into causal pathways, our analytical approach enables the development of testable hypotheses to better delineate the intersection of reproductive maturation and psychological health. Second, biochemical analytes were measured across three core laboratories. Our choice of laboratories was deliberate in that total testosterone was measured using liquid chromatography mass spectrometry by a laboratory approved by the Center for Disease Control Hormone Standardization Program and consistent with recommendations put forth by the latest International Guidelines for PCOS (4). Of note, all analytes were measured consistent with other adolescent research from our research group (46) to improve comparability of findings across studies. This study also had limitations which should guide future research. These include a small sample size and cross-sectional study design; without following the participants, we were unable to ascertain which youth went on to develop PCOS versus those who did not. In addition, this study, much like current research on psychopathology in PCOS, measured psychological symptoms and endocrine markers at a single, non-fasting and random time point and therefore cannot offer insight whether there might be cyclical variations in mood, emotion, and behavior consistent with hormonal fluctuations. Future studies should include serial assessments to measure daily changes in psychological symptoms with fluctuations in reproductive endocrinology. Finally, participant self-report of Tanner Staging, hirsutism, and acne may reflect youth perceptions and experiences but not perfectly agree with physician assessment (47–49). For these reasons, we view our study as a preliminary one, requiring replication in larger, prospective samples.

Whether or not psychopathology and PCOS are entangled early in the disease trajectory matters. Earlier onset of psychopathology tends to predict recurrent and more severe mental health challenges and a greater likelihood of developing major depressive episodes in the face of comparatively small amounts of life stress (50, 51). As the chronicity of depression, and anxiety are linked with treatment adherence and eligibility for interventions for PCOS, adolescent onset of psychopathology holds repercussions both for the severity of PCOS symptoms and comorbid disease management (52–54). Psychological disorders tend to be more malleable in adolescence than adulthood (55), presenting a window of opportunity for effective intervention historically overlooked in PCOS practice. Our results suggest that endocrine indicators of PCOS and correlates of dysfunction are connected to depression and anxiety early in adolescence, raising important questions about disease pathogenesis and new directions for research and clinical management.

## Data Availability

The de-identified data supporting the conclusions of this article will be made available by the authors, upon reasonable request and per institutional guidelines.
