# Supplementary material for: Psychological symptoms in perimenarcheal adolescents: association with PCOS risk factors
Source: Front Endocrinol (Lausanne). 2025 Jun 12;16:1551958. doi: 10.3389/fendo.2025.1551958 (PMC12197959; doi:10.3389/fendo.2025.1551958)
Supplement: Supplementary file 1 [file DataSheet1.docx]

Supplementary Material

# Supplementary Table

# Supplementary Table 1

| **Variable** | **Data Structure** | **Comments** |
| --- | --- | --- |
| **General Demographics** |  |  |
| Age (years) | Continuous |  |
| Race | Categorical | White  Non-White |
| Ethnicity | Categorical | Hispanic  Non-Hispanic |
| Family History of PCOS or Infertility (Infx) | Categorical | Parent/guardian reported history of PCOS or difficulty getting pregnant up to and inclusive of grandparents |
| Psych Meds | Categorical | Yes or No; the participant has ever taken medications that affect psychological symptoms |
| Highest Parental Education | Ordinal | Highest level of education achieved by any parent/guardian in the household.  1 – 1^st^ to 5^th^ grade  2 – 6^th^ to 8^th^ grade  3 – 9^th^ to 12^th^ grade  4 – High school graduate; GED equivalent  5 – Associate’s degree  6 – College graduate  7 – Advanced degree |
| **Physical Experience of Puberty** |  |  |
| Reproductive Stage | Categorical | Pre-menarcheal  Post-menarche (<1 year)  Post-menarche (1-2 years) |
| Reproductive Category | Categorical | Regular  Irregular  *Post-menarcheal participants only* |
| Sexual Maturity Scale (SMS) of Pubic Hair | Continuous | Participant self-reported (perceived) sexual maturity stage. |
| Sexual Maturity Scale (SMS) of Breast Development | Continuous | Participant self-reported (perceived) sexual maturity stage. |
| Acne | Continuous | Acne grading scale (0 through 4) |
| Dysmenorrhea | Categorical | No dysmenorrhea  Mild dysmenorrhea  Moderate dysmenorrhea  *Post-menarcheal participants only* |
| **Body Habitus** |  |  |
| Waist Circumference (WC) | Continuous |  |
| Waist-Hip-Ratio (WHR) | Continuous |  |
| BMI Percentile | Continuous | BMI for age and height |
| **Symptoms or Features of PCOS** |  |  |
| AMH (ng/mL) | Continuous |  |
| Free Androgen Index (FAI, %) | Continuous |  |
| Sex Hormone Binding Globulin (SHBG) | Continuous |  |
| LH:FSH Ratio | Continuous |  |
| Total Testosterone | Continuous |  |
| Hirsutism Score (revised*) | Continuous | The initial score conducted at Weill Cornell included vellus hairs in the score. Revised refers to the systematic removal of body hair vellus scores to obtain a revised Hirsutism Score from the original score. Regional scores with hair type descriptions were systematically documented at this site. |
| HbA1c | Continuous | Measure for insulin resistance |
| **Endocrine Features of Puberty** |  |  |
| Estradiol (pg/mL) | Continuous |  |

## Supplementary Figures


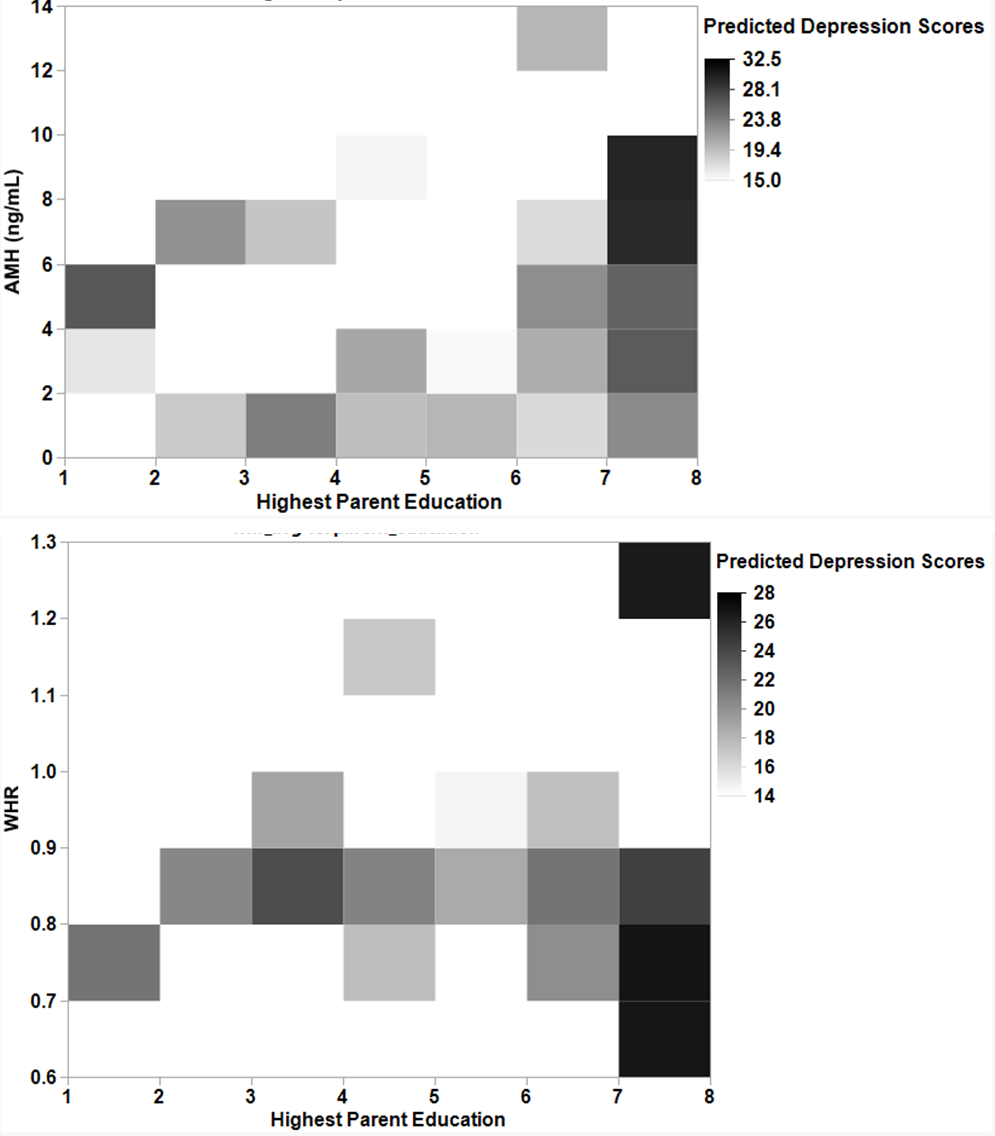


**Supplementary Figure 1.** Heat maps depicting the inter-relationships between Highest Parent Education by WHR and AMH by Parent Education on predicted depression scores generated from the random forest plots. It is important to note for interpretation that other variables in the full dataset contribute to the predicted scores and modify the x- and y- relationships on the outcome (depression). In other words, at Parent Education 1-2, the relationship between parent education and AMH on predicted depression is not independent of the other variables.


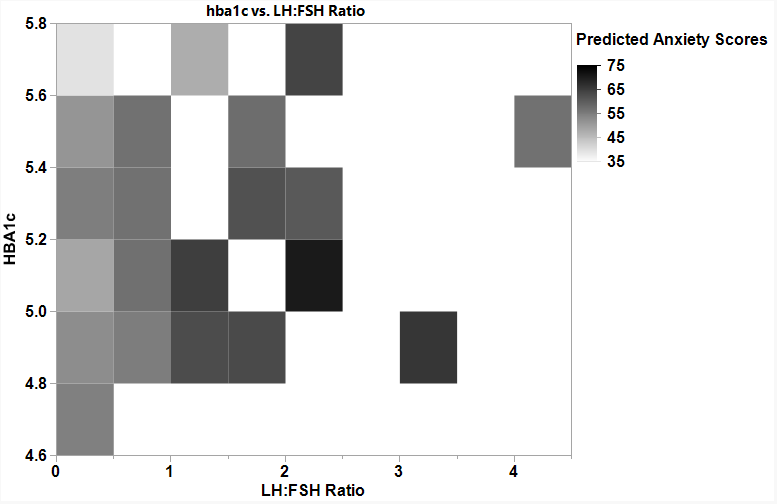

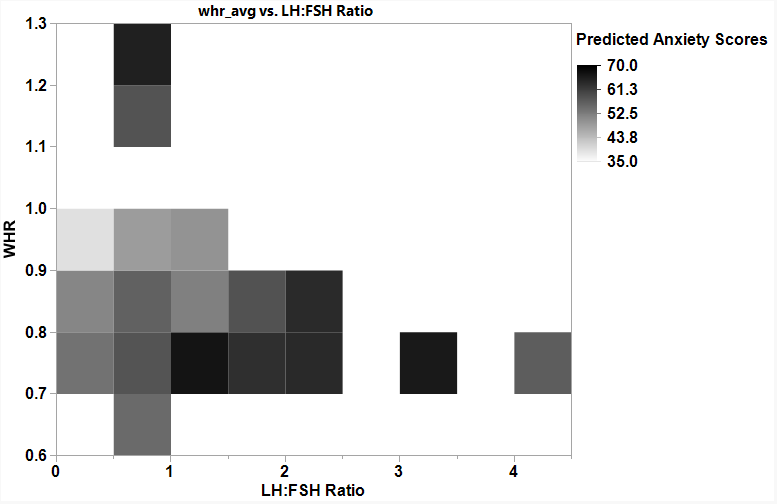


**Supplementary Figure 2.** Heat maps depicting the inter-relationships between LH:FSH Ratio, WHR, and HbA1c on predicted anxiety scores generated from the random forest plots. It is important to note for interpretation that other variables in the full dataset contribute to the predicted scores and modify the x- and y- relationships on the outcome (anxiety).


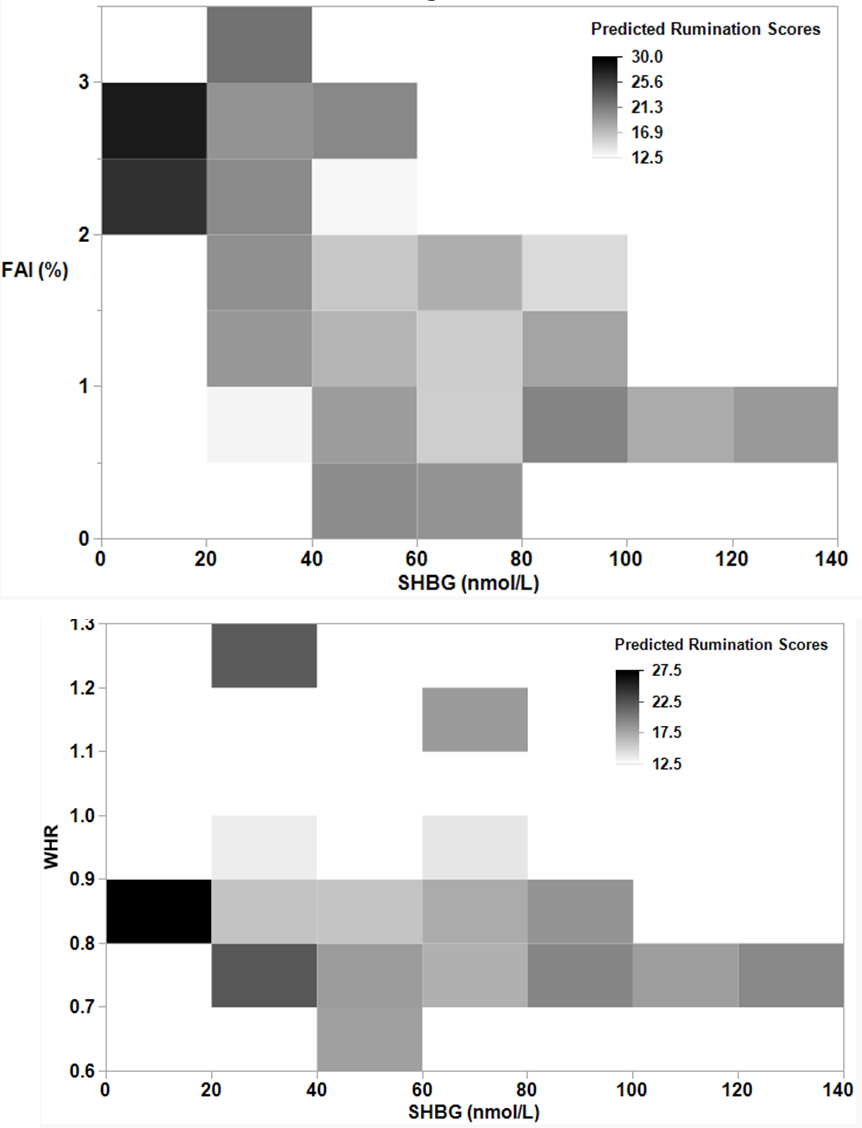


**Supplementary Figure 3.** Heat maps depicting the inter-relationships between SHBG, FAI, and WHR on predicted rumination scores generated from the random forest plots. It is important to note for interpretation that other variables in the full dataset contribute to the predicted scores and modify the x- and y- relationships on the outcome (rumination).

**
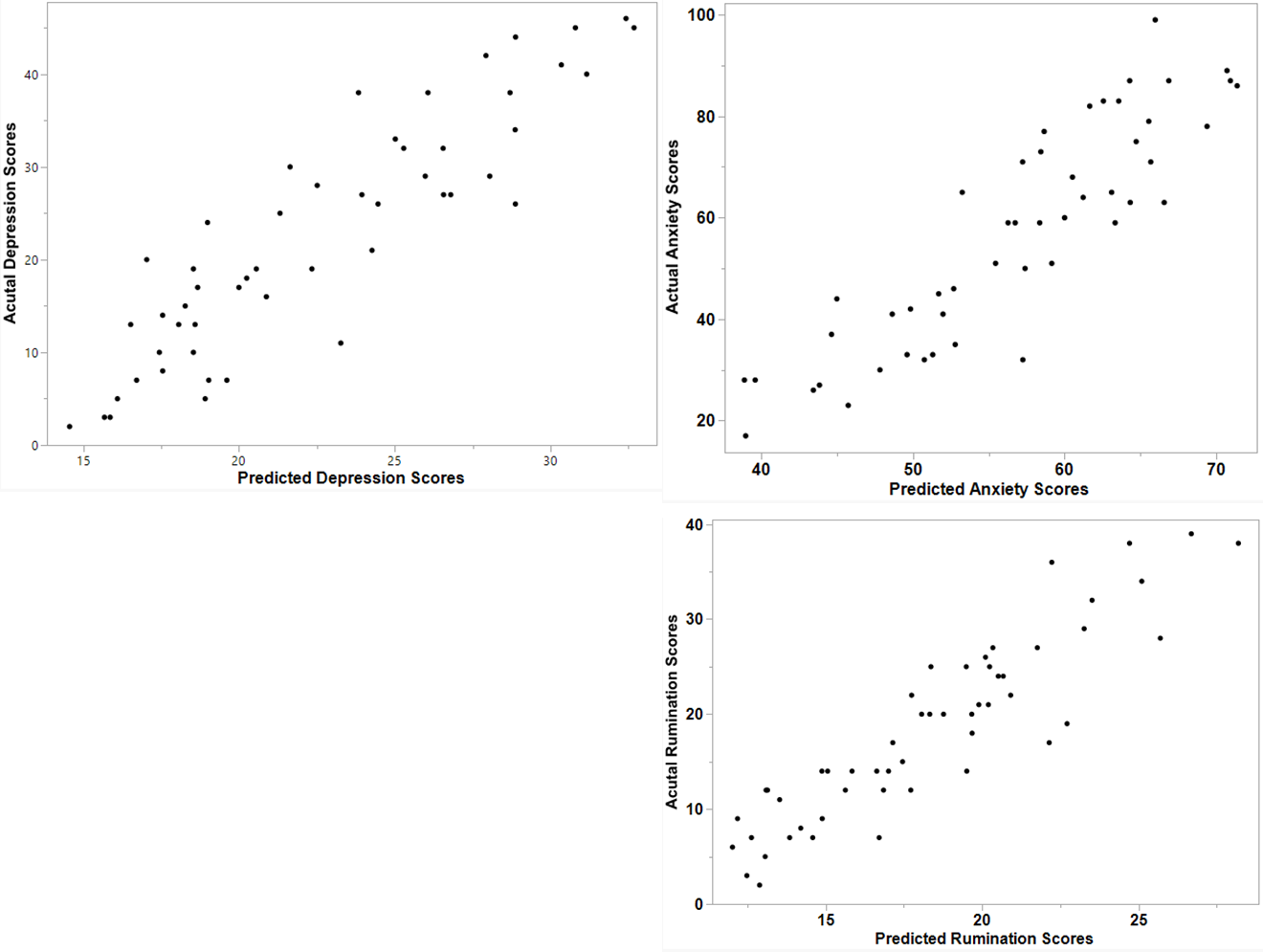
**

**Supplementary Figure 4.** Actual (participant report) versus predicted (generated from Random Forest Plots) depression, anxiety, and rumination scores. Heatmaps in Supplementary Figures 1-3 are generated from predicted values.
